# Development of an Air Pneumatic Suspension System for Transtibial Prostheses

**DOI:** 10.3390/s140916754

**Published:** 2014-09-09

**Authors:** Gholamhossein Pirouzi, Noor Azuan Abu Osman, Azim Ataollahi Oshkour, Sadeeq Ali, Hossein Gholizadeh, Wan A. B. Wan Abas

**Affiliations:** 1 Department of Biomedical Engineering, University of Malaya, Kuala Lumpur 50603, Malaysia; E-Mails: azuan@um.edu.my (N.A.A.O.); sadeeqcpo@um.edu.my (S.A.); gholizadeh@um.edu.my (H.G.); drirwan1@um.edu.my (W.A.B.W.A.); 2 Department of Mechanical Engineering, University of Malaya, Kuala Lumpur 50603, Malaysia; E-Mail: azim.ataollahi@siswa.um.edu.my

**Keywords:** air pneumatic system, semiconductor pressure sensor, socket design, pressure distribution, socket-stump interface, adjustability

## Abstract

The suspension system and socket fitting of artificial limbs have major roles and vital effects on the comfort, mobility, and satisfaction of amputees. This paper introduces a new pneumatic suspension system that overcomes the drawbacks of current suspension systems in donning and doffing, change in volume during daily activities, and pressure distribution in the socket-stump interface. An air pneumatic suspension system (APSS) for total-contact sockets was designed and developed. Pistoning and pressure distribution in the socket-stump interface were tested for the new APSS. More than 95% of the area between each prosthetic socket and liner was measured using a Tekscan F-Scan pressure measurement which has developed matrix-based pressure sensing systems. The variance in pressure around the stump was 8.76 kPa. APSS exhibits less pressure concentration around the stump, improved pressure distribution, easy donning and doffing, adjustability to remain fitted to the socket during daily activities, and more adaptability to the changes in stump volume. The volume changes were adjusted by utility of air pressure sensor. The vertical displacement point and reliability of suspension were assessed using a photographic method. The optimum pressure in every level of loading weight was 55 kPa, and the maximum displacement was 6 mm when 90 N of weight was loaded.

## 1. Introduction

Artificial limbs enable amputees to engage in normal daily activities. The suspension system in lower limb prostheses has a vital role in prosthetic function [1–5]. Prosthetists recommend a proper suspension system for the amputee according to the circumstances of the patient. The residual limb-socket interface is filled with a liner and is in direct contact with the skin and socks. The liner works as a cushion for the residual limb and alleviates shock from the contact between the prosthesis and the residual limb. Several systems are employed to secure the stump inside a socket and connect the suspension system to the pylon (adaptor) and the foot. These systems include the belt and suprapatellar cuff [6], figure-of-8 belt [7], sleeve suspension [8], supracondylar-suprapatellar suspension [9], supracondylar suspension, thigh corset silicon liner suspension, and distal locking pin, lanyard, and suction suspension [10,11]. However, silicon and polyethylene foam liners are the most common suspension systems used. Modern biofeedback and pressure measurement instruments are used to record the most effective pressure under dynamic and static conditions [12–14]. Several techniques such as computer-aided design, computer-aided manufacture, and finite element method are also used to gain more information and reduce the fabrication time of sockets [15–17].

However, currently available suspension systems are accompanied by several problems that are linked to the continuous change in residual limb size, volume, donning, and doffing, especially in systems with a vacuum liner [18,19]. In other words, the socket, liner, and residual limb should be in full contact to ensure proper pressure distribution [20]. The continuous change in volume or size of the residual limb leads to the loss of contact and interruption of pressure distribution in the entire system [21]. Consequently, the pressure is concentrated on some parts of the system, which results in injury to the residual limb [22]. The problem created by size changes is traditionally solved by adjusting to the change, such as by wearing socks [23]. Dissatisfaction of patients with their prostheses is mainly due to strains and injuries associated with socket mismatch, which result from changes in the stump size and form. The shuttle-lock system/pin liner also creates a large suction distally on the residual limb and causes chronic skin change [24]. Meanwhile, a misaligned pin in the pin-lock suspension system introduces difficulties such as failure in locking the suspension system to the pylon and blisters on a residual limb of the amputee when the pin suspension system becomes older and is improperly fitted [25]. Therefore, prosthetic designers have introduced new locking systems, such as vacuum and magnetic systems.

Issues related to the control of pressure distribution caused by the continuous change in residual limb size, donning, and doffing of new designs remain unsolved despite attempts to address these limitations. Therefore, the present study was designed to develop a new air pneumatic suspension system (APSS) by aid of semiconductor pressure sensor to overcome residual limb volume changes and eliminate problems related to the pin-lock and vacuum locking systems.

## 2. Experimental Section

### 2.1. Particulars of the Design

**Figure 1.**
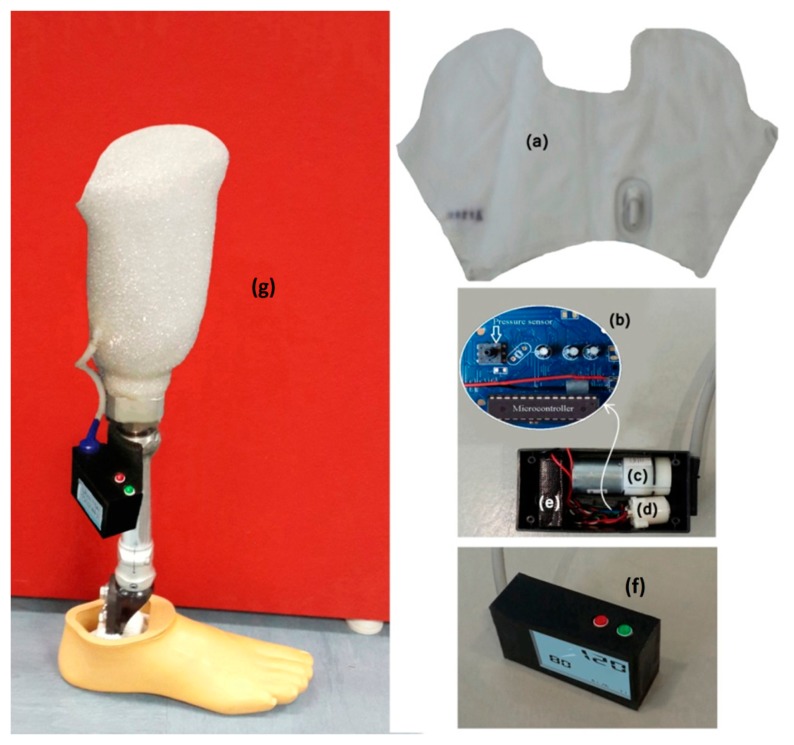
Feature and components of the APSS: Bladder (**a**); Control circuit board (including a pressure sensor and microcontroller) (**b**); Pump (**c**); Valve (**d**); Battery (**e**); Operation system (**f**); Assembled transtibial prostheses (**g**).

**Figure 2.**
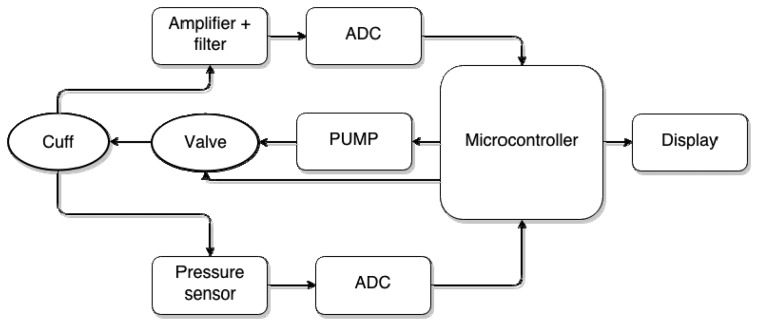
System operation chart.

Current suspension systems were replaced with the newly developed APSS. The APSS comprises a control board with microcontroller that includes a semiconductor pressure sensor (ADP41410/ Panasonic, USA), an air cuff attached inside the socket, air pumps, and pressure-regulating valves (Figure 1). The desired pressure between the socket and the stump is defined by the user and controlled smartly by an APSS microcontroller (Figure 2).

To use the new pneumatic system, the amputee first defines the desired pressure before placing the stump into the socket. Then, the amputee presses the control key to initiate the air pumps, which pump air into the bladder until the desired pressure value is reached. Afterward, pressure is maintained at a constant value. System operation is controlled and guaranteed through standard protocols of multiple feedbacks by the microprocessor and pressure data transmission through the module that contains the sensor. Finally, to doff the prosthesis, the amputee needs to press a button to release the air pressure. The stump will easily come off the socket.

### 2.2. Participants

The APSS was tested on five patients (Table 1). The median of height, stump length, and mass of the patients were 176 cm (SD = 4.82), 13.5 cm (SD = 0.95), and 73 kg (SD = 4.82), respectively. The average height, mass, and stump length were 174.81 cm, 76.4 kg, and 13.6 cm, respectively. The inclusion criteria were no ulcers and wound in the stump, ability to walk without assistive devices and stump length of at least 13 cm (from inferior edge of patella to distal end). It was a requirement that the participants be experienced prosthetic users (more than 2 years). Amputations were caused by motorbike accidents for three participants and vascular disease for the other two. Their levels of activity varied from K2 to K3 based on the Medicare Functional Classification Level [26]. The Ethics Committee of the University of Malaya Medical Centre approved this study, and informed written consents were obtained from all the subjects.

### 2.3. Experimental Protocol

**Figure 3.**
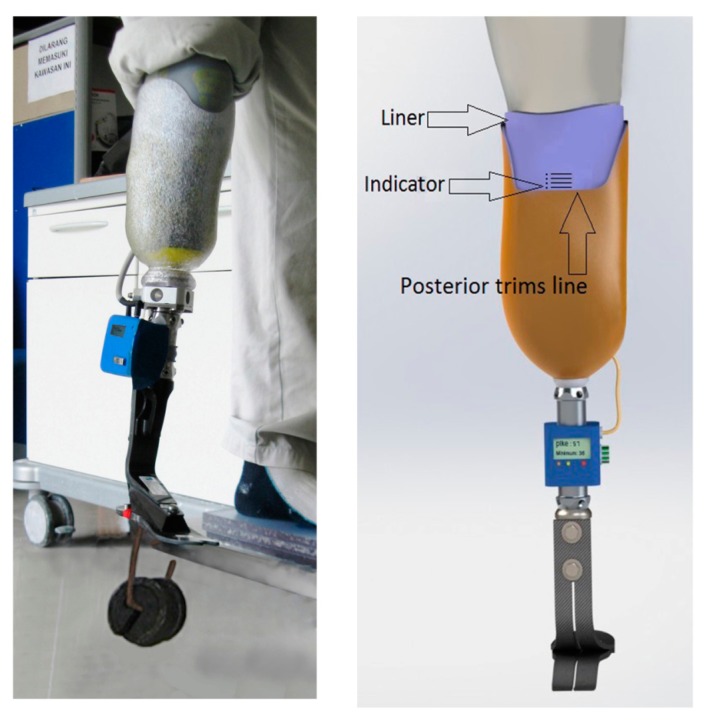
Pistoning within the socket was monitored to measure the vertical displacement point.

To evaluate the performance of the APSS, transtibial prostheses with the Iceross silicon liners were used, but the pins were removed from the liners. A certified prosthetist was recruited to control and check the stumps as well as to reassemble and align the prostheses. The patients were then asked to wear the prostheses, and oral reports from the patients were recorded. The subjects practiced walking in the motion analysis laboratory under the control of the prosthetist who aligned the prostheses according to their needs. After that, subjects were free to walk around to become familiar with and adapt to the new prosthetic devices. The subjects wore the new system for 5 hours before attending the motion laboratory for pressure measurements. In order to assess pistoning, gait was mimicked by using different loads and pressure; we varied the prosthesis load as well as pressure [27,28]. Pistoning within the socket was monitored to measure the vertical displacement point, beginning with the pressure of 25 kPa. A load weight of 30 N was added and was repeated up to 90 N. The displacements were recorded for each prosthesis and then this procedure was repeated for the pressure of 30 kPa up to 60 kPa. A photographic method was used for this measurement [29]. The indicator attached to the liners was used as a measuring reference for the displacement from the edge of the posterior trim line of the socket (Figure 3). Before loading the weights, the patients were asked to stand bearing full weight on the prosthetic limb. Then, pressure and loading weights were applied under the non-weight bearing condition. Under each condition, photographs were taken from a fixed distance using a photo stand.

**Figure 4.**
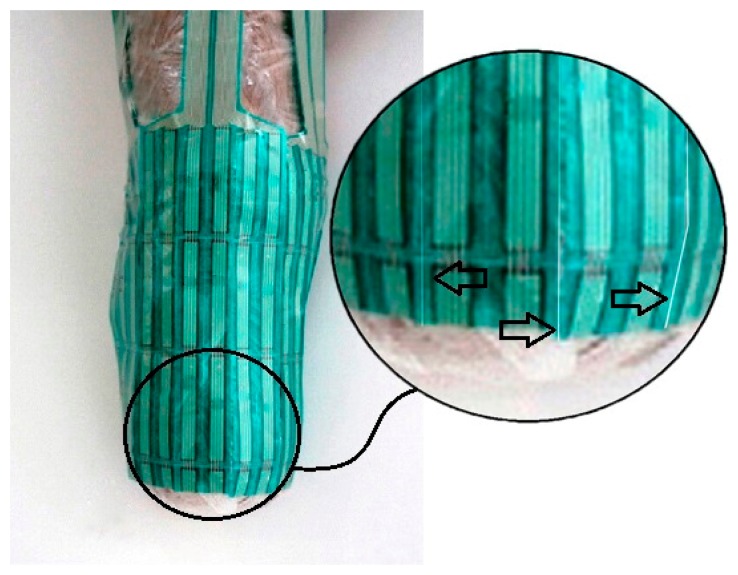
Transducers were divided in parts to fit the contours of liners.

Next, the subjects were asked to walk at a self-selected speed on a walkway that was 8 m long and 4 m wide. Four pressure sensors were attached to the anterior, posterior, medial and lateral surfaces of the residual limb. A F-Scan (Tekscan, South Boston, MA, USA) pressure measurement system was used to evaluate the pressure at all the surfaces [30]. To reduce the chances of inaccuracies pressure sensors arrays were equilibrated and calibrated. To equilibrate, the sensors arrays were placed inside the Tekscan PB100T pressure bladder and subjected to a repeated pressure of 100 kPa, based on the manufacturer's instructions. The calibration was accomplished based on each subject's body weight. The applied pressure for calibration was the ratio of each subject's body weight to the respective sensor area [31]. We follow the same protocol for all the subjects. The residual limbs were covered with cellophane plastic wrap, and each transducer was attached to the cellophane plastic wrap with spray adhesive (Scotch Super Adhesive, 3M Corporate, St. Paul, MN, USA) to ensure that the transducer was appropriately positioned on the stump. Each pressure sensor was individually trimmed to fit to the contours of the residual limb, this arrangement allow covering more than 95% of a residual limb (Figure 4). Data were obtained for 12 s with a sampling rate of 50 Hz. Four consecutive trials were completed by the subjects on the walkway, and approximately eight to nine steps were taken in each trial. The middle step of each trial was selected. Mean peak pressures of four trials were used for statistical analyses.

### 2.4. Pressure Distribution between Socket-Stump Interface

**Figure 5.**
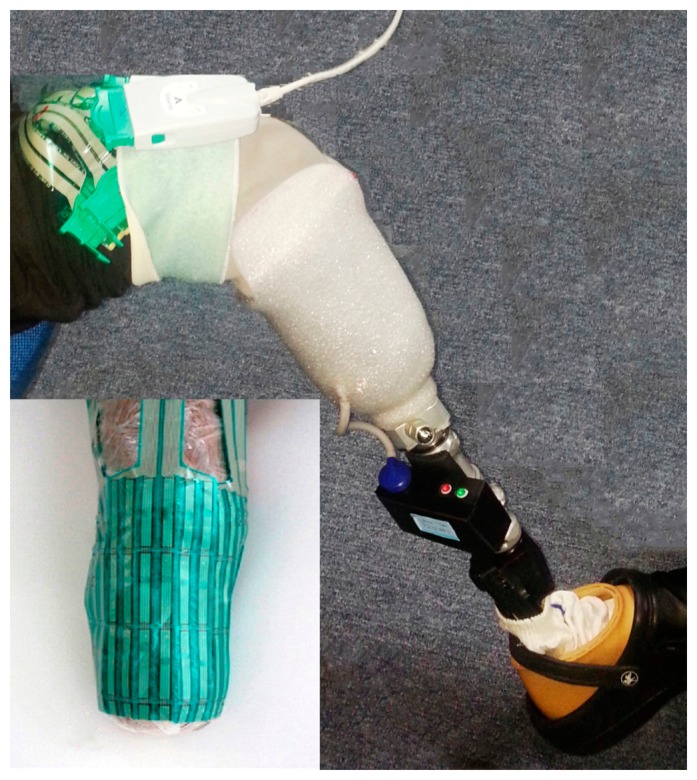
F-Scan test setup.

**Figure 6.**
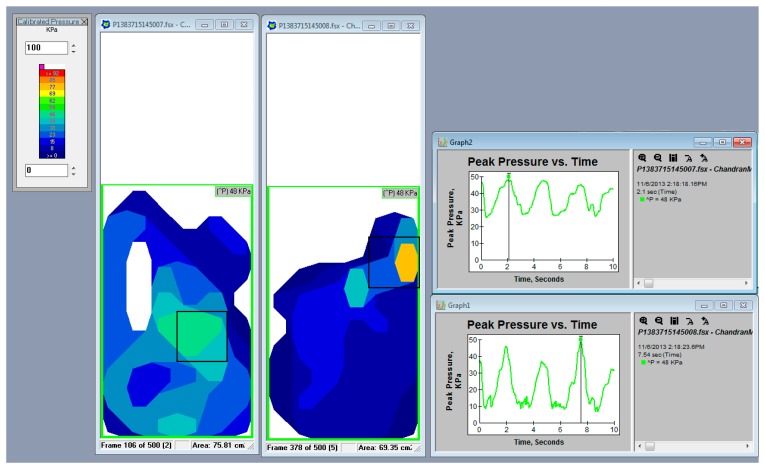
Data and graphs show pressure distributing and the pick points of pressure.

Pressure distribution between the stump and the new suspension was tested using F-Scan sensors, as shown in Figures 5 and 6. Pressure values were measured in 12 regions of the residual limb.

## 3. Results

### Applicability as a Suspension System

**Figure 7.**
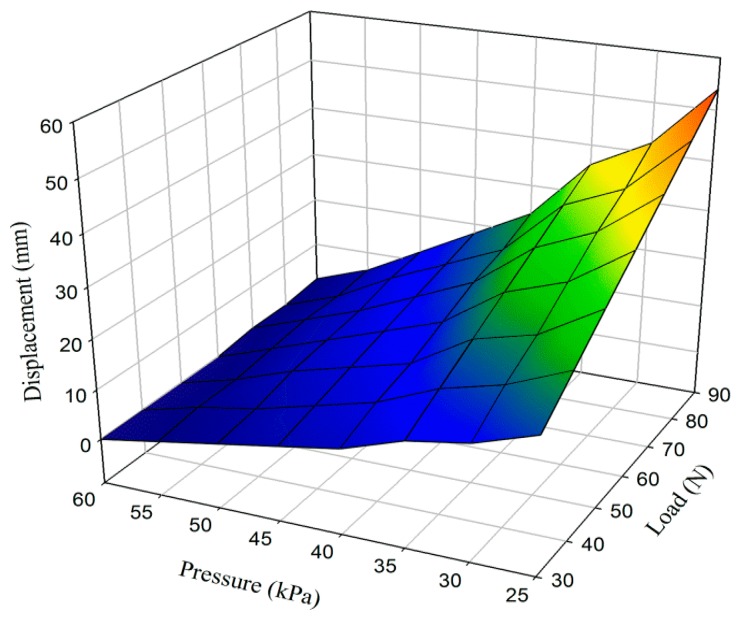
Average values of distances between posterior trims line and liner (mm).

The applicability of the APSS was evaluated by assessing the amount of pistoning. The needed pressure to maintain contact between the stump and the artificial limb without any applied external load was approximately 10 kPa. Different pressures were exerted on the stump and the socket. External weight was applied on the artificial limb, and pistoning was measured for different cases. Results are presented in Figure 7.

The acceleration factors of the dynamic acceleration tolerance of the system were evaluated by replacing the force loading under static condition presents the pattern of data points. The data points follow a linear pattern. This result indicates a strong negative relationship between pressure exerted by inflation bladder and load applied. Pistoning clearly decreased when pressure was increased by the inflation bladder in the socket-stump interface. On other hand, displacement showed increasing trend by the increase in external load.

The pressure was read at the distal, middle, and proximal portions of the stump in the anterior, posterior, lateral, and medial sides. Average pressure was 54 kPa on the anterior and posterior sides, and 45.6 kPa on the lateral and medial sides (Table 2).

## 4. Discussion

Pressure distribution in the socket–stump interface and socket fit, which are the main indicators for evaluating a socket [1,32], were considered in this study. A photographic method [29] and F-scan were used to assess pistoning and pressure distribution, respectively. The complete removal of pistoning is neither possible nor desirable, although 10 mm or less pistoning is acceptable and make sense of balance and confidence to the amputees [33]. In the new suspension system, size can be adjusted, and volume changes are compensated by the intelligent system. An adjustable socket is important in various daily activities [23]. In actual walking, an artificial leg has different rates of acceleration and needs different levels of stability, and these differences can be addressed by the APSS pressure controller during the passive phase. Performed tests show that pressure can be adjusted for normal conditions and greater mobility, if necessary. The APSS showed a better pressure at the different locations. The mean peak pressure (kPa) at the anterior in medial for APSS is 56.43 kPa, while in a recent study conducted by Ali *et al.* [34] this pressure for a Dermo Liner and Seal-In X5 Liner were 62.7 kPa and 86.5 kPa, respectively. Studies conducted on the biomechanical behavior of the stump and the socket interface [13,35–41] highlight the importance of the fitting. The APSS also can provide a better fitting because after inflation pressure the bladder mimicked stump geometry and lay on it. Another advantage of the APSS system is the prosthesis fitting adjustment will perform after donning and doffing will perform by release pressure. Therefore, donning and doffing is easer in the APSS system than in the current sockets. It should be noted that the pneumatic system was employed in previous prosthesis as “Pump it up” and “Air Cushioned” systems. However, both “Pump it up” and “Air Cushioned” systems are not specialized as suspension system and cushioning is their main concept. Although, based on the manufacturer comment [42], these system can play a role of suspension system only in special cases and pin and lock still are remain there. The main problem with these systems is creating stress concentrations at points of the stump and also shear stress increases in small sections due to pistoning. In counter the basis of the concept of APSS is a suspension system and cushioning is its fringe benefits. In addition, automatic pressure adjustment is unique capability of the APSS and is not is not available in aforementioned systems. Meanwhile, based on the previous works conducted by Staros [43] the maximum weight of the prosthesis should be in range of 2.5–2.9 kg. The average weight of the prostheses with air pneumatic suspension system (APSS) employed in present work was about 2.2 kg.

## 5. Conclusion

The APSS was introduced in this study as a new prosthesis suspension system. The system adapts to the daily changes of stump volume and provides enhanced fit. Furthermore, less pistoning and easier donning and doffing result from its use.

## Figures and Tables

**Table 1.** Demographics of the participants.

**Subjects**	**Gender**	**Age**	**Amputation Causes**	**Level of Activities**	**Height (cm)**	**Mass (kg)**	**Stump Length (cm)**
1	Male	36	Motorbike accident	K3	172	85	15
2	Male	72	Vascular disease	K2	180	73	13.5
3	Male	50	Motorbike accident	K3	176	65	13
4	Male	23	Motorbike accident	K3	168	60	13
5	Male	38	Vascular disease	K3	178	99	16

Limited community ambulator (K2), Community ambulator (K3).

**Table 2.** The average peak pressure (kPa) for all sensors sites at the medial and lateral anterior, posterior, residual limb.

**Anterior**			**Posterior**		
	
**Proximal (Var.)**	**Middle (Var.)**	**Distal (Var.)**	**Proximal (Var.)**	**Middle (Var.)**	**Distal (Var.)**

54.81 (13.21)	56.43 (7.69)	51.3 (9.56)	50.94 (12.41)	56.52 (6. 82)	53.73 (17.26)

**Medial**			**Lateral**		
	
**Proximal (Var.)**	**Middle (Var.)**	**Distal (Var.)**	**Proximal (Var.)**	**Middle (Var.)**	**Distal (Var.)**

42.84 (8.83)	44.91 (8.91)	44.55 (6.23)	47.7 (12.05)	50.49 (7.52)	43.38 (13.25)
